# Are LMICs Achieving the Lancet Commission Global Benchmark for Surgical Volumes? A Systematic Review

**DOI:** 10.1007/s00268-023-07029-x

**Published:** 2023-05-16

**Authors:** Priti Patil, Priyansh Nathani, Juul M. Bakker, Alex J. van Duinen, Pranav Bhushan, Minal Shukla, Samir Chalise, Nobhojit Roy, Anita Gadgil

**Affiliations:** 1 Department of Statistics, BARC Hospital, Mumbai, 400094 India; 2 Department of Surgery, Hinduhridaysamrat Balasaheb Thackeray Medical College, Dr. Rustom Narsi Cooper Municipal General Hospital, Mumbai, India; 3 Department of Public Health and Nursing, Norwegian University of Science and Technology (NTNU), Trondheim, Norway; 4 Clinic of Surgery, St. Olav’s Hospital, Trondheim University Hospital, Trondheim, Norway; 5 Department of Public Health, Institute of Global Public Health, University of Manitoba, Winnipeg, Canada; 6 Department of Maternal Health, UNICEF, Bhopal, India; 7 Department of Global Public Health, Karolinska Institute, 171 77 Stockholm, Sweden; 8 The George Institute for Global Health, New Delhi, India; 9 Department of Surgery, BARC Hospital, Mumbai, 400094 India

## Abstract

**Introduction:**

The Lancet Commission on Global Surgery (LCoGS) set the benchmark of 5000 procedures per 100,000 population annually to meet surgical needs adequately. This systematic review provides an overview of the last ten years of surgical volumes in Low and Middle- Income-Countries (LMICs).

**Methodology:**

We searched PubMed, Web of Science, Scopus, Cochrane, and EMBASE databases for studies from LMICs addressing surgical volume. The number of surgeries performed per 100,000 population was estimated. We used cesarean sections, hernia, and laparotomies as index cases for the surgical capacities of the country. Their proportions to total surgical volumes were estimated. The association of country-specific surgical volumes and the proportion of index cases with its Gross Domestic Product (GDP) per capita was analyzed.

**Results:**

A total of 26 articles were included in this review. In LMICs, on average, 877 surgeries were performed per 100,000 population. The proportion of cesarean sections was found to be high in all LMICs, with an average of 30.1% of the total surgeries, followed by hernia (16.4%) and laparotomy (5.1%). The overall surgical volumes increased as the GDP per capita increased. The proportions of cesarean section and hernia to total surgical volumes decreased with increased GDP per capita. Significant heterogeneity was found in the methodologies to assess surgical volumes, and inconsistent reporting hindered comparison between countries.

**Conclusion:**

Most LMICs have surgical volumes below the LCoGS benchmark of 5000 procedures per 100,000 population, with an average of 877 surgeries. The surgical volume increased while the proportions of hernia and cesarean sections reduced with increased GDP per capita. In the future, it's essential to apply uniform and reproducible data collection methods for obtaining multinational data that can be more accurately compared.

## Introduction

In 2015, the World Health Assembly passed a resolution (WHA 68.15) declaring emergency and essential surgical and anesthesia care as critical components of Universal Health Coverage (UHC) [1, 2]. An estimated 11–32% of the global disease burden is due to surgically correctable illnesses [3–5]. The Lancet Commission on Global Surgery (LCoGS) has created a framework to evaluate and set targets for access, delivery, and financial impact of surgeries needed to address the global burden of surgical diseases. Surgical volume is one of the indicators mentioned by LCoGS, which captures a country's met surgical need, with a benchmark of 5000 procedures per 100,000 population annually in 2030.

Many countries, especially low- and middle-income countries (LMICs), are facing a high burden of communicable diseases and an increasing burden of non-communicable and surgical diseases like cancers and road traffic injuries. The poorest countries, which have one-third of the global population, have only 6% of the 313 million surgeries worldwide taking place each year [6]. Some countries report that less than 10% of the total surgical need are being met [7, 8]. To find the gaps between the proposed LCoGS indicators and the current surgical care delivery, data and literature on surgical volumes and performed operations are crucial.

Many LMICs have challenges reporting the LCoGS indicators. Many LMICs lack appropriate mechanisms to estimate the surgical volume on a regular basis. Studies have reported that using hernia and the cesarean section as proxy indicators to assess the surgical capacity as a more comprehensive enumeration of all operations performed may not be feasible in many LMICs [9, 10].

Though surgical diseases and delivery of care by performing essential operations have been in focus since 2015, literature on volumes of surgical procedures performed in LMICs has been sparse. So, we considered the research question, how many studies have been published from low and middle-income countries, on the volumes of surgeries performed, and how many have addressed the proxy indicators of surgical system capacity like cesarean section, laparotomy, and hernia? With this research question, we aimed to review the existing literature from LMICs on the volumes of surgeries performed and the proportion of proxy indicators like hernia and cesarean sections to the total surgical volumes. We also aimed to analyze the correlation of the Gross Domestic Product (GDP) per capita of these countries to the surgeries performed in these LMICs that reported the surgical volumes. In addition, we have documented the limitations and barriers of data collection in various settings.

## Material and methods

For this systematic review, we searched the PubMed, Web of Science, Scopus, Cochrane, and EMBASE databases for the publication period from the year 2011 to 2021. The Population–Concept–Context strategy was used to extract the articles using a combination of terms mentioned in Table 1.

**Table 1:** Population–Concept–Context guided search strategy components

Population	The patient belongs to the age group > = 18 years	
Concept	Surgical volume (concept 1)	Surgical procedures, Operative (MeSH), Surgical needs, Emergency surgeries, Essential surgeries, Surgical Enumeration, Surgical volume, Global Surgery, Surgical burden, Surgical Care, Surgical delivery, Bellwether Procedures, Operations
	Index surgeries (concept 2)	Hernia, Cesarean Section, Laparotomy
Context	LMIC (concept 3)r2	Names of all the LMIC countries
Excluded search words	NOT terms (concept 4)	Pediatric surgery, Neonatal surgery, Infants, Childbirth, Vaginal delivery, Maternal mortality, Neonatal mortality, Abortion, Contraceptive method, Surgical site infection, Randomized control trial, Clinical trial, Case–control trial, Therapeutics trial
Search strategy	((concept 1) AND (concept 2) AND (concept 3)) NOT (concept 4)	

Preferred Reporting Items for Systematic Reviews and Meta-Analyses (PRISMA) checklist were used for reporting this systematic review [11], and the study was registered in the International Prospective Register of Systematic Reviews (PROSPERO) (Registration number CRD42022336158).

Surgical procedures were defined as procedures performed in an operation theater requiring local, general, spinal, or regional anesthesia [6, 12]. We included studies conducted in LMICs that gave information about the volumes of surgery, either by enumeration through community-based surveys, district/ state or country-level hospital records data, or by estimating surgical volumes through mathematical modeling.

### Inclusion/exclusion criteria

Studies that collected and presented surgical volume were included. The LCoGS has identified cesarean sections, laparotomies, and open fracture treatment as the Bellwether procedures [6]. However, challenges with the definition and collection of the open fracture treatment procedure have been described [13]. Previously, inguinal hernia repair and cesarean section were identified as proxy indicators for overall surgical volume [9]. Therefore, we selected cesarean section, laparotomy, and inguinal hernia repair as index surgeries which were extracted from the included articles where available.

Studies based on high-income countries were beyond the scope of this systematic review. We excluded studies describing surgical education or training of medical professionals, postoperative mortality, morbidity, or complications. Further, we also excluded articles that described overall surgical capacity or disease burden without mentioning operative volumes. Single institutions-based studies were included only if they described surgeries performed at district or higher-level hospitals.

### Review process

**Fig. 1:**
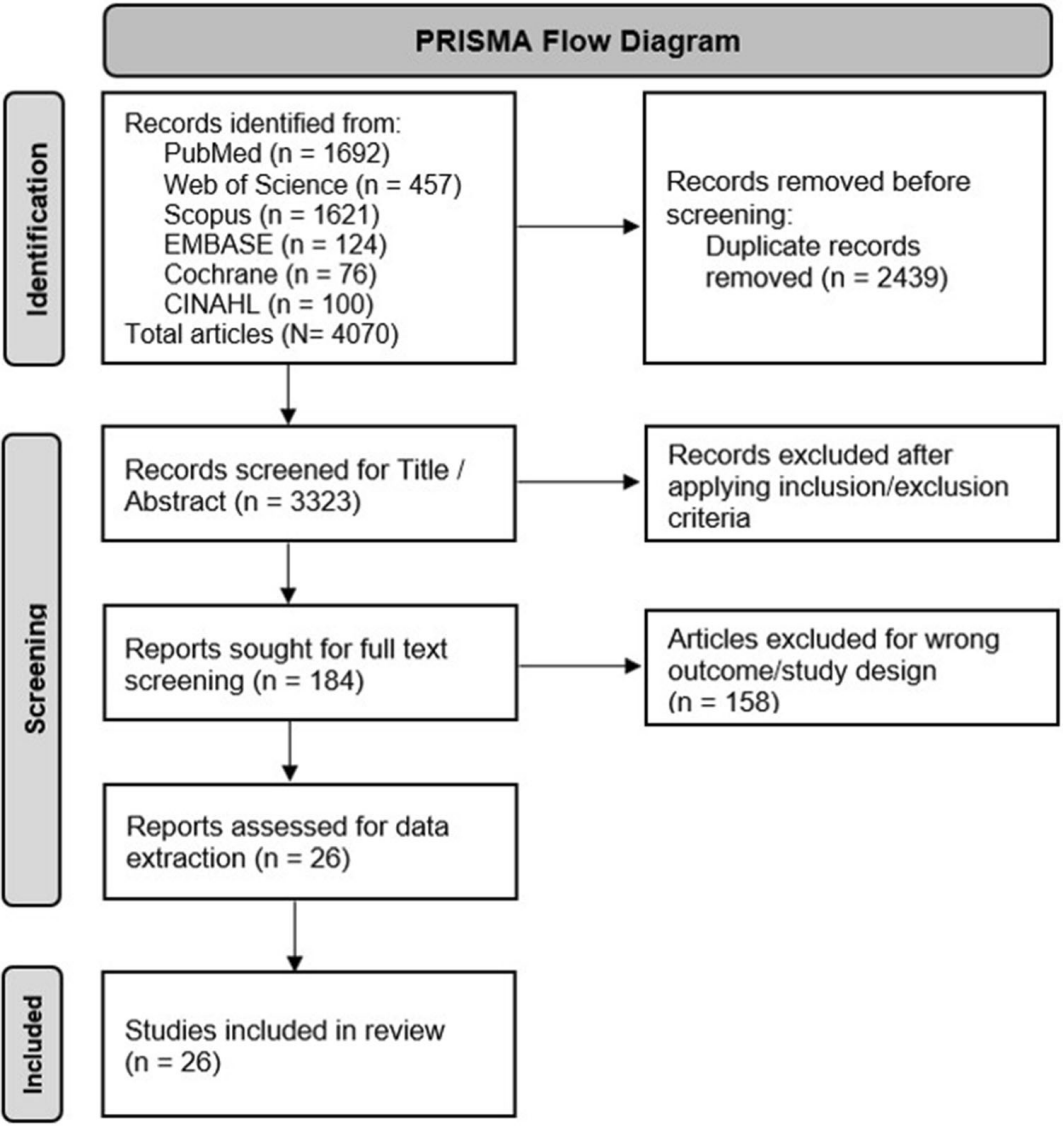
PRISMA diagram

We have identified 4070 articles from initial search. All the identified studies were entered in Rayyan QCRI [14], an intelligent-systematic-review online platform developed and maintained by the Qatar Computing Research Institute (QCRI), and duplicates were removed. After removing duplicates, 3323 articles were selected for title/abstract screening. A two-stage screening process was followed; two reviewers independently screened the articles for inclusion, and a third reviewer made the final decision in case of conflicts. Articles not fulfilling the inclusion/exclusion criteria (3139) were excluded. Further shortlisted 184 articles were examined for full text, and 158 of those were excluded for the wrong outcome (other than total surgical volume or index surgery-related data). Finally, 26 studies were included for final data extraction and analysis (Fig. 1).

Selected articles were distributed among all reviewers for data extraction. Based on the literature review and research question, a data coding file was prepared in Microsoft Excel® (Microsoft, Redmond, Washington, USA) database. A pilot data extraction was initially done for two studies by all the reviewers. Discrepancies and queries were discussed on a weekly call between all the contributing reviewers, and necessary adaptations were made in the final data extraction sheet. The extraction sheet contained article characteristics like study duration, setting, and type. Further, it consisted of the study outcome, index surgeries volume and their proportions to total surgical volumes if available, the total volume of surgeries per 100,000 population, country estimates if available, interventions to increase the volume of the surgeries, and any barriers to data collection.

### Data analysis

Surgical volumes per 100,000 population were calculated based on the reported number of surgeries and population estimates. For articles reporting population estimates, these numbers were included. For articles that did not report the population estimates, the World Bank data on the population during the study year were considered to calculate the surgical volume per 100,000 population estimates [15]. The average volume was calculated for further analysis for countries with multiple studies. Further, the proportions of index surgeries to the total number of surgeries performed in a study setting were estimated. The association between the country's surgical volumes and proportions of index surgeries with its GDP per capita [16] during their respective study periods was assessed. The statistical analysis was conducted using R studio and Microsoft Excel® (Microsoft, Redmond, Washington, USA).

## Results

In total, 26 studies that satisfied the inclusion criteria were included in the final analysis. Of these studies, 21 (81%) were conducted in Africa. Half of the studied articles were from Ghana, Uganda, and Sierra Leone. Ten studies (38%) were based on national-level data, and nine (35%) were from multicenter data. Total surgical volume was documented in 19 of 26 (73%) articles, while in the remaining 7 (27%) studies, volumes of at least one or more index surgeries were calculated. Study characteristics are presented in Table 2.

**Table 2:** Article characteristics (Total included articles 26)

Description	# Studies
*Country*	
Ghana	5
Sierra Leone	4
Uganda	4
Liberia	2
India	2
New Guinea	2
South Africa	1
Zambia	1
Sub-Saharan Africa	1
Nigeria	1
Brazil	1
Ethiopia	1
Niger	1
*Data collection points*	
National data	10
Multi-center study	9
Single-center study	4
Community-based data	2
Systematic review	1
*Study setting*	
Public	16
Public and private	6
Private	1
Not available	3
*Study period*	
< 12 Months	5
1—5 Years	15
> 5 Years	1
Not available	5
*Study type*	
Observational	24
Interventional	1
Systematic review	1
*Outcome of interest*	
Total surgical volume with either or all index surgeries	13
Total surgical volume	6
Only volumes of one or more index surgeries	7

Table 3 outlines the surgical volume and proportion of index surgeries to the total surgeries at the country level. Overall, an average of 877 surgeries per 100,000 population was estimated. Only two studies from India and Brazil have documented volumes of surgeries close to the LCoGS benchmark. Cesarean section was the most commonly performed surgery among the three index surgeries in the studied articles, representing an average of 30.1% of the total surgeries. The Indian study by Bhandarkar P et al. documented that 3.8% of the surgeries were cesarean sections [17]. In contrast, it was the most commonly done surgery in Sub-Saharan Africa, accounting for 63.0% of all surgeries [18]. On average, inguinal hernias comprised 16.4% of the total surgeries, with a range of 2.8% in India and 30.0% in Sub-Saharan Africa [17, 18]. Laparotomies accounted for 5.1% of the total surgeries, ranging from 0.8 to 7.5% between studies.

**Table 3:** Low-middle-income country-wise surgical volume per 100,000 population and percentages of index surgeries to total surgeries

Study country	Reference	Study period	GDP^^^	Surgical volume*	Cesarean section %	Hernia %	Laparotomy %
India	Bhandarkar P et al. [17]	2017	1980.7	5259	3.8	2.8	–
	Ensor T et al. [36]	2015	1605.6	280	–	–	–
Brazil	Massenburg B et al. [21]	2015	8814.0	4433	–	–	–
Ghana	Abdullah F et al. [37]	2008	1217.1	728	14.6	10.8	0.8
	Ohene-Yeboah M et al. [38]	2016	1972.0	–	–	13.0	–
	Gyedu A et al. [39]	2014	2012.3	869	–	7.5	–
	Gyedu A et al. [40]	2014	2012.3	869	27.0	8.0	4.2
	Gyedu A et al. [41]	2014	2012.3	–	27.0	–	–
New Guinea	Stokes MAR et al. [42]	2013	2729.9	783	–	–	–
	James K et al. [43]	2012	2790.7	606	–	–	–
Sub-Saharan Africa Liberia	Grimes CE et al. [18]	2012	1827.3	581	63.0	30.0	14.0
	Joharifard S et al. [44]	2018	710.3	–	31.6	22.3	6.5
	Adde H et al. [7]	2017	721.1	462	43.1	21.0	4.1
	Joharifard S et al. [44]	2016	740.9	–	37.2	29.1	4.5
Sierra Leone	Lindheim-Minde B. et al. [8]	2017	496.7	372	27.6	16.0	2.5
	Bolkan HA et al. [45]	2014	714.7	288	–	–	–
	Lindheim-Minde B. et al. [8]	2012	566.4	400	20.2	21.5	4.3
	Bjerring AW et al. [9]	2012	566.4	400	20.2	21.5	–
	Bolkan HA et al. [46]	2012	566.4	400	21.0	22.4	4.6
South Africa	Tefera A et al. [47]	2015	6259.8	–	56.0	–	–
Zambia	Cheelo M et al. [48]	2012	1763.1	188	–	–	–
Nigeria	Anderson JE et al. [49]	2017	1968.6	169	–	–	–
Uganda	Albutt K et al. [50]	2017	746.8	–	46.6	–	7.3
	Albutt K et al. [51]	2016	736.7	145	55.8	5.0	7.5
	Löfgren J et al. [52]	2011	832.6	186	17.8	3.7	1.6
	Ajiko M et al. [53]	2010	822.5	–	10.0	–	4.6
Ethiopia	Reshamwalla S et al. [54]	2010	341.5	123	–	–	–
Niger	Sani R et al. [55]	2007	390.3	–	19.2	27.5	–
Overall average			877.0	30.1	16.4	5.1	

^ GDP per capita of a country considered for the study data collection period, * Surgical Volume per 100,000 population

**Fig. 2:**
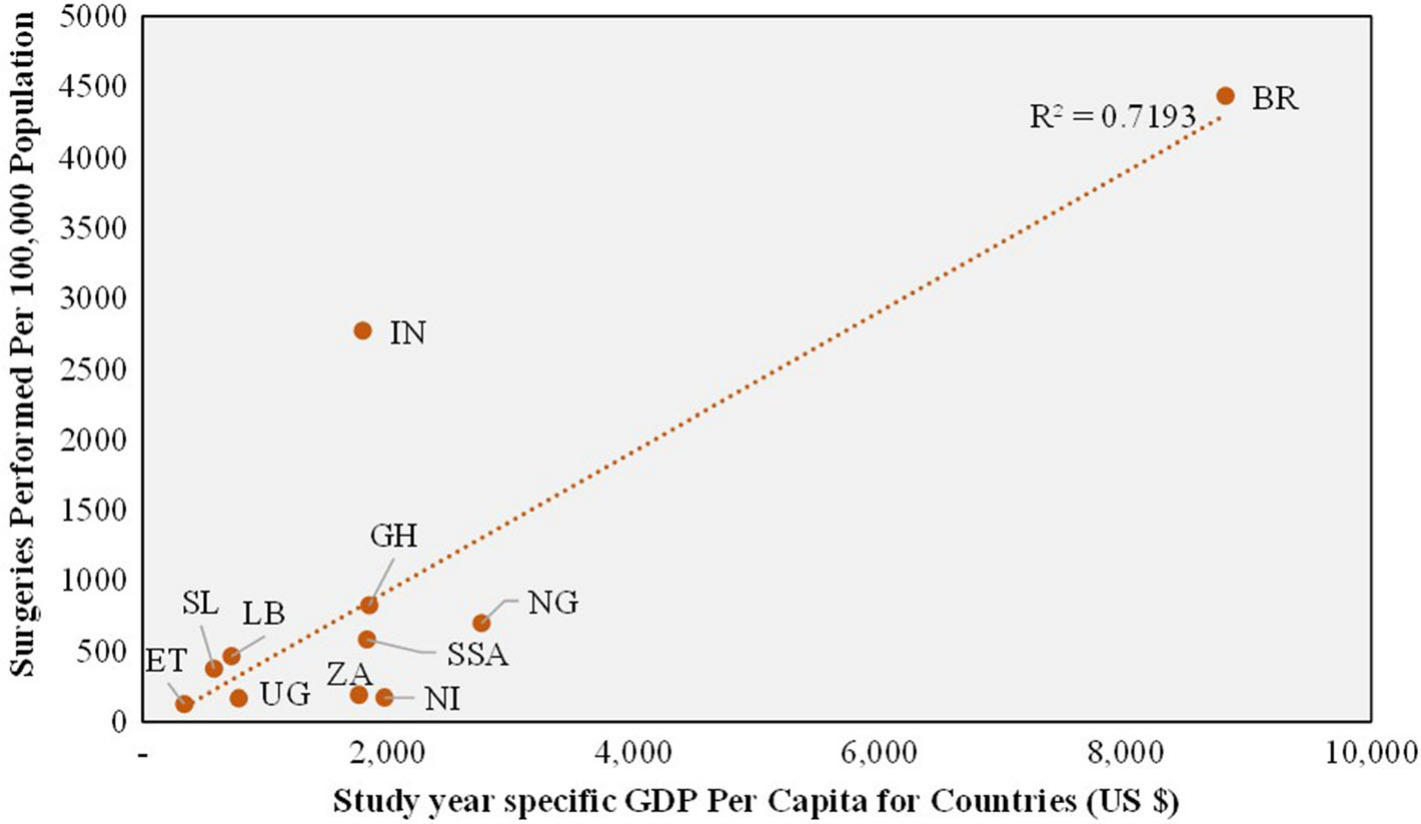
Association of surgical volume with per capita GDP

Figure 2 describes the association of average surgical volumes with the GDP per capita of the countries. The overall surgical volume increased as the GDP per capita of the country was higher, with a strong association (*R*^2^ = 0.72). Brazil (4433 surgeries per 100,000 population) has shown the highest surgical volume per 100,000 population among the studied articles, followed by India (2770 average surgeries per 100,000 population). The study from Ethiopia reported the least number of surgeries (123 per 100,000 population).

**Fig. 3:**
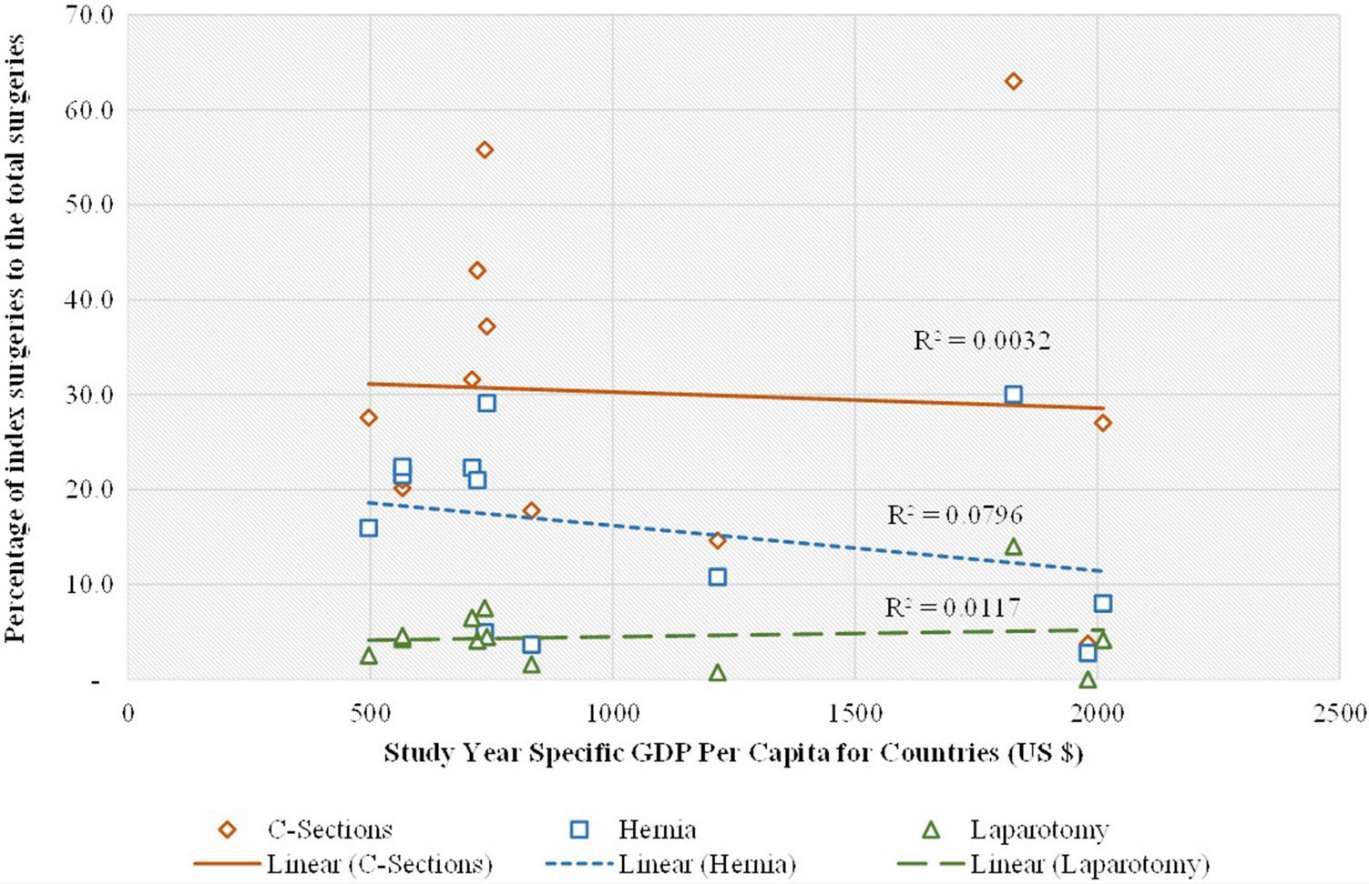
Comparison of percentage of index surgeries out of total surgeries with study year-specific GDP per capita in LMICs

Figure 3 indicates a negative association between GDP per capita in a country and the proportion of hernias performed out of the total surgical volume (*R*^2^ = 0.0796). To a lesser extent, a similar decrease is seen in the proportions of cesarean sections (*R*^2^ = 0.0032). A slight increase was seen in the proportion of laparotomy as the GDP per capita increased. (*R*^2^ = 0.0117).

### Barriers and limitations to data collection for surgical volumes indicator

Nine of 26 studies discussed barriers to data collection for surgical volumes in the country. The main barriers to data collection were inconsistent nomenclature, reporting, and procedures excluded from the enumeration. Procedures performed outside the operation theater and camp settings were not included in articles from Ghana and India. Similarly, in some studies, procedures performed outside the district or public hospitals were not accounted for. In countries where the private sector contributes mainly to the surgical services provided, excluding these would mean the loss of a many procedures. A study from Zambia reported that remote locations of hospitals prevented data reporting and collection. Studies from Ghana and Sierra Leone documented that, where the specialist workforce is unavailable, non-specialist workers perform surgeries. However, provider-specific data on procedures were often not available.

## Discussion

We found that none of the LMICs met the benchmark for the surgical volume of 5000 surgeries per 100,000 population. Brazil with higher GDP than other studied countries and India’s study from a community under UHC were approaching the LCoGS benchmark. In addition, countries with higher GDP per capita had higher surgical volumes per 100,000 population. The proportion of index surgeries, cesarean sections, and hernias within total surgeries tended to decrease for countries with higher GDP per capita.

National surgical volumes are vital for determining the country’s surgical capacity and developing national plans to increase it. The LCoGS benchmark is based on important health indicators: a life expectancy of 74–75 years, a maternal mortality ratio of less than or equal to 100, and a minimum estimated surgical capacity to match the prevalence of surgical disease in each country [6, 19, 20]. Based on our review, we cannot conclude whether this benchmark is ideal for all settings. We estimated an average of 877 surgeries per 100,000 population in LMICs. This is remarkably low compared to the LCoGS benchmark, indicating a significant unmet surgical need in LMICs. Only Brazil [21] and a study performed in a specific Indian setting [17]. under the UHC scheme is comparatively closer to the benchmark.

We correlated the surgical volume with the country's GDP per capita. A systematic review reported that the surgical volume of elective and non-elective surgeries significantly decreased due to the economic crisis. An economic downturn was associated with increased patients experiencing severe adverse outcomes, such as an increased risk of death, for non-elective/emergency surgeries [22]. A study reported an increase in cesarean sections during economic decline [23], which was likely due to the prioritization of emergency procedures. According to research from the USA, there is a substantial positive association between cosmetic surgery procedures and the GDP per capita, demonstrating that as GDP rises, so do the types of surgeries performed beyond emergency cases [24, 25]. Sierra Leone experienced a dramatic decline in surgical volume from 2012 to 2014, despite a rise in GDP per capita, which may be attributed to the country's Ebola epidemic in 2014 [26], highlighting the dynamic nature of the surgical volume indicator. Holmer et al. discovered that health expenditure was the significantly correlated factor for surgical volumes when compared the association between different economic factors and surgical volume [27]. Weiser and colleagues developed the model to estimate the number of procedures. They described health expenditure as the explanatory variable to predict surgical volume [28]. Health expenditure is the percentage of the size of the overall economy. It is shown that when the GDP per capita increases, the percentage of health expenditure also increases leading to increased surgical volumes [29]. We considered the country’s overall economy instead of health expenditure to analyze its association with surgical volumes. For national healthcare system development, it is vital to look at the causes of the economic downturn and how it affects surgical interventions, patients, and doctors. The distribution of healthcare resources and future surgery policy planning may benefit from being aware of these patterns.

Among the three index surgeries, the proportion of cesarean sections and hernia decreased slightly as GDP per capita increased. In contrast, there is a slight increase trend seen in laparotomies with rising GDP per capita. We observed some trends in the proportion of index surgeries but have insufficient data to make conclusions about the correlations. The cesarean section to total operations (C/O) represents local surgical capacity, as most, if not all, of the cesarean sections, will be done by local surgeons and are prioritized by the health systems and local governments. Studies have used the C/O ratio to estimate surgical volume [10, 30]. Caesarean sections and hernia operations account for the majority of surgeries at the low end of the GDP per capita, whereas other procedures are more frequently performed at the high end of the GDP per capita where surgical resources are more readily available.

There is considerable heterogeneity in the methodology used to evaluate surgical volume. Data collection varied based on the settings, like district hospitals, military, and private surgical providers. Being labor- and resource-intensive, the methods of estimation range from mathematical modeling to household surveys. Estimating the number of surgeries to the population using surgeon interviews may be effective in smaller countries with greater surgeon-to-population ratios and fewer hospitals but not in densely populated countries with low surgeon-to-population ratios, such as India. The most commonly used definition for a surgical procedure is a procedure executed in an operating theater with some form of anesthesia. This definition, however, poses some challenges. Many essential surgeries, like chest tube insertion and tracheostomy, are performed in emergency rooms or at patient’s bedside without the use of general or regional anesthesia. Due to varying definitions of what constitute surgery, these are not counted as surgeries. Some high-volume surgeries are performed in camp settings, which is a common practice in LMICs [31]. This makes the surgical volume indicator challenging to collect and interpret. Furthermore, surgeries, like laparoscopic surgeries for asymptomatic gallstones, cesarean sections, and so on, are performed more often as a result of advanced technologies, fewer side effects, and the fear of complications [32, 33]. This could be masking the true figure of unmet surgical need.

There is a need for a set of standardized methods for collecting surgical volume data that are quantifiable, practicable, and reproducible in each country's context. Each method has its own advantages and disadvantages, and the detailed discussion is material for a separate and independent methodological paper. The overarching goal of collecting data based on indicators is to evaluate and promote surgical system improvement. Indicators are critical in translating data into action and holding accountable the responsible stakeholders to advocate for improved surgical care and the improvement of the country's surgical plans. However, the difference in methodology makes comparing results across geographic areas challenging. Given the cost-effectiveness of surgical interventions in LMICs [34], investing in surgery can help save millions of lives while improving overall economic development [35].

### Strengths and limitations

We used more recently available articles from the LMIC facilities with or without mathematically modeled data to present the surgical volumes. The main challenge in calculating surgical volume rate is the lack of a universally acceptable definition of surgical procedures. The collection of surgical volume-related data is restricted in resource-limited settings. Studies have captured data from various sources like household surveys, operating theater logbooks, and national registries. Several facility, performer, and population-level barriers are documented in research that falls outside the focus of this study. While data collection challenges are evident in government settings, addressing the barriers to volume study in the private sector, which contributes significantly, have received less attention. For example, despite providing up to half of the health care in Sub-Saharan Africa, the private and private-not-for-profit sectors account for only 29.8% of the facilities reviewed [18]. We found that only 7 of the 26 (27%) papers included the private sector, which may have understated both the reported surgical volume as well as the private sector’s contribution to the surgical volume analysis. We did not consider variations in surgical volumes within each country, such as comparing urban and rural areas of countries in our study. We did not examine open fracture fixation as an index surgery, despite its status as a bellwether procedure, due to a lack of research on its use as a proxy indicator for surgical volume. Open fracture reduction surgeries, including trauma-related procedures, are considered more complex and require more resources than many other surgical procedures. These types of surgeries performed in LMICs can be highly variable depending on various factors, such as the burden of disease, the availability of resources, and the presence of specialized expertise. Therefore, the open fracture surgery may not accurately reflect the total surgical volume in LMICs. Studies with open fracture as a proxy indicator for surgical volume are surprisingly rare. This subgroup of studies revealed the percentage contribution of cesarean sections, laparotomies, and hernias with greater confidence. A significant proportion of surgical procedures performed in LMICs are likely to be for pediatric patients. However, there is often a lack of data resources on pediatric surgical procedures in LMICs, limited access to surgeries due to inadequate healthcare infrastructure, inadequate resources, and a shortage of trained healthcare professionals. This can make it challenging to accurately estimate the volume of pediatric surgeries. So, this study has not included pediatric surgeries separately for estimating surgical volume. Furthermore, we considered the GDP per capita of the country specific in the year when the particular studies were conducted, which may have overlooked the policy changes that occurred during that time. A set of robust, reproducible data collection methods is required to collect multinational data at better comparable time points in the future. Recently the UTSTEIN consensus process involved global experts to clearly define the surgical indicators highlighted by the LCoGS. The panel recognized the need for collecting granular data on surgical indicators like volumes. The consensus statement also acknowledged there would be a trade-off between granular data and the difficulty in collecting this by all the countries globally [12].

## Conclusion

Most LMICs, with an average surgical volume rate of 877 surgeries per 100,000 population, did not reach the benchmark set by the LCoGS. There was heterogeneity in describing or enumerating surgeries, methods of estimation, and denominators for the proportion of index surgeries. There is a need for a standardized data collection method for surgical volumes. The total number of surgeries performed per 100,000 population increased as the GDP increased. Including data from The World Bank indicators of surgery can improve our understanding of surgical indicators, determining their correlation, utility, and accuracy of reporting and collecting.
